# Selectable Markers and Reporter Genes for Engineering the Chloroplast of *Chlamydomonas reinhardtii*

**DOI:** 10.3390/biology7040046

**Published:** 2018-10-10

**Authors:** Lola Esland, Marco Larrea-Alvarez, Saul Purton

**Affiliations:** 1 Department of Life Sciences, Imperial College London, South Kensington Campus, London SW7 2AZ, UK; l.esland17@imperial.ac.uk; 2 School of Biological Sciences and Engineering, Yachay-Tech University, Hacienda San José, Urcuquí-Imbabura 100650, Ecuador; malarrea@yachaytech.edu.ec; 3 Institute of Structural & Molecular Biology, University College London, Gower Street, London WC1E 6BT, UK

**Keywords:** *Chlamydomonas reinhardtii*, chloroplast engineering, selectable markers, reporter genes

## Abstract

*Chlamydomonas reinhardtii* is a model alga of increasing interest as a cell factory for the production of valuable compounds, including therapeutic proteins and bioactive metabolites. Expression of foreign genes in the chloroplast is particularly advantageous as: (i) accumulation of product in this sub-cellular compartment minimises potential toxicity to the rest of the cell; (ii) genes can integrate at specific loci of the chloroplast genome (plastome) by homologous recombination; (iii) the high ploidy of the plastome and the high-level expression of chloroplast genes can be exploited to achieve levels of recombinant protein as high as 5% total cell protein; (iv) the lack of any gene silencing mechanisms in the chloroplast ensures stable expression of transgenes. However, the generation of *C. reinhardtii* chloroplast transformants requires efficient methods of selection, and ideally methods for subsequent marker removal. Additionally, the use of reporter genes is critical to achieving a comprehensive understanding of gene expression, thereby informing experimental design for recombinant applications. This review discusses currently available selection and reporter systems for chloroplast engineering in *C. reinhardtii*, as well as those used for chloroplast engineering in higher plants and other microalgae, and looks to the future in terms of possible new markers and reporters that will further advance the *C. reinhardtii* chloroplast as an expression platform.

## 1. Introduction

Since the development of recombinant DNA technology several decades ago, genetically engineered organisms have become an increasingly popular source of industrially-valuable proteins and metabolites. Whilst most current applications exploit bacteria or yeasts, notably *Escherichia coli* and *Saccharomyces cerevisiae*, or mammalian cells, microalgae such as the unicellular green alga *Chlamydomonas reinhardtii* are emerging as alternative expression systems for a number of recombinant products [1,2,3]. *C. reinhardtii* has been utilised extensively as a laboratory model for over 50 years, aiding the understanding of photosynthetic processes, flagella biogenesis and function, and circadian rhythms (reviewed in [4]). The alga is particularly suited to molecular–genetic studies as the nuclear, mitochondrial and chloroplast genomes have been sequenced [5,6], and transformation procedures exist that allow for the integration of exogenous DNA into all three genomes [1,7]. *C. reinhardtii* possesses a single, large chloroplast harbouring 50–80 copies of the circular, ~206 kb chloroplast genome (or ‘plastome’) that contains 99 genes encoding core components of the photosynthetic apparatus and the organelle’s transcription–translation apparatus [6]. The chloroplast represents an attractive site for synthesis of recombinant products, not least because the genetic system is simple and highly active, and the organelle is the site of numerous biosynthetic pathways. Whilst foreign genes could be introduced into the nuclear genome such that their protein products are targeted into the chloroplast, there are several key advantages for engineering the plastome directly [8]. Firstly, genes-of-interest (GOIs) can be targeted to specific loci within the plastome via homologous recombination, allowing for precise and predictable manipulations. Secondly, much higher levels of protein accumulation can be achieved through chloroplast expression compared to nuclear expression. Thirdly, the chloroplast stroma appears to support the correct folding and disulfide bond formation of recombinant proteins [9], and any cytotoxic effects of the proteins is minimised by their strict confinement to the chloroplast [10]. Such engineered algal strains offer the potential for low-cost phototrophic production in which high-value products, such as therapeutic proteins and bioactives, are produced from CO_2_ and simple nutrients through photosynthesis [2,3]. Furthermore, *C. reinhardtii* has GRAS (‘Generally Recognised as Safe’) status with no evidence of toxic or mutagenic components within the cell [11]. It therefore offers, for certain applications, the possibility of circumventing extensive and costly purification processes, for example, for oral or topical applications. 

A large number of recombinant proteins have been successfully produced in the *C. reinhardtii* chloroplast using the established transformation methods [12]. The focus has been primarily on therapeutic proteins that do not perturb the biology of the chloroplast [13]. These include livestock vaccines, such as a potential foot-and-mouth vaccine [14], and, more recently, one that may protect against avian flu [15]. Candidate human vaccines have also been expressed, against malaria [16], *Staphylococcus aureus* infection [17], and tumours caused by human papilloma virus [18]. The latter two have been shown to cause an immune response and confer some level of protection in mice trials [17,18]. Chloroplast engineering has also been used to produce other therapeutically-valuable proteins, such as antibodies [9], a class of antibody–drug conjugates known as immunotoxins that have promise in cancer treatment [10], and recombinant allergens for the treatment of peanut and birch pollen allergies [19,20]. Furthermore, recent research has shown the viability of expressing a toxin known to kill mosquito larvae in the *C. reinhardtii* chloroplast, an anti-mosquito strategy that could prove particularly useful due to the cohabitation of *C. reinhardtii* and mosquito larvae in certain habitats [21]. Additionally, microalgae are being explored as industrial biotechnology platforms for the production of metabolites that have applications ranging from biofuels to bioactives [22]. Such metabolic engineering requires further advances in the molecular toolkit, allowing precisely regulated expression of multiple transgenes. These tools are still at an early stage of development and require the application of synthetic biology approaches. However, the next few years are likely to witness significant advances in this field [23], as we are already seeing for the metabolic engineering of plant chloroplasts [24].

**Figure 1:**
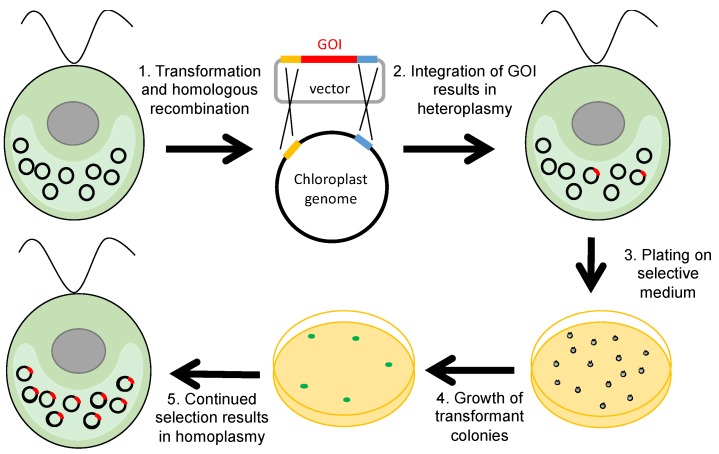
*C. reinhardtii* chloroplast transformation. The gene of interest (GOI) is integrated into some of the copies of the plastome by homologous recombination. Only cells containing at least one copy of the transgene and selectable marker will be able to survive on the selective medium, and will form colonies. At this stage it may be necessary to perform multiple rounds of single colony selection to attain homoplasmy.

DNA delivery into the *C. reinhardtii* chloroplast is achieved by bombarding a lawn of cells with DNA-coated gold microparticles using a particle gun [11] or, less efficiently, agitation of a suspension of a cell wall-deficient strain and DNA with glass beads [25]. Initially, the integration of the DNA into the polyploid plastome results in a heteroplasmic mixture of untransformed and transgenic plastome copies within a single chloroplast. The acquisition of the GOI by every copy of the plastome—homoplasmy—is subsequently achieved by the application of selective pressure, as cells replicate such that progeny containing more transgenic copies are favoured (Figure 1).

**Figure 2:**
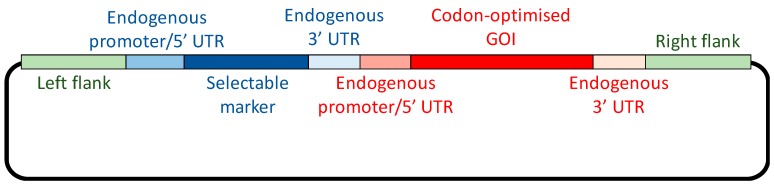
Transformation plasmid. A schematic of the required elements of a *C. reinhardtii* chloroplast transformation vector. UTR = untranslated region.

Introducing the GOI into the plastome represents the first stage, but the design of the GOI is critical to its successful expression. Typically, the *cis* elements required for transcription and translation are derived from highly expressed endogenous genes, such as those encoding the core subunits of the photosynthetic complexes. As illustrated in Figure 2, the coding sequence is fused to a strong promoter, together with the 5′ and 3′ untranslated regions (UTRs) that are required for transcript stability and translation initiation. These three elements can be derived from the same endogenous gene or separate genes: for example, the *rrnS* gene encoding the 16S RNA has the strongest promoter in the chloroplast, and this can be combined with a 5′ UTR from a highly expressed protein-coding gene such as *atpA* [26]. However, gene expression in the chloroplast differs from that of bacterial systems in that regulation occurs principally at the level of translation initiation [27]. It is therefore important to combine a strong promoter with a 5′ UTR that is not tightly controlled by endogenous feedback mechanisms, or requires sequence elements immediately downstream of the start codon for efficient translation [25].

A second important consideration in the design of transgene constructs is that the *C. reinhardtii* plastome is AT-rich (approximately 64% AT), and displays a strong bias towards AT-rich codons. Consequently, codon optimisation of transgenes often results in improved translation efficiency, and hence higher levels of protein accumulation [28]. Software tools are available for the design of codon optimised synthetic genes, including “Codon Usage Optimizer” developed in the Purton lab [29], and many of the successfully expressed recombinant proteins produced in the chloroplast have been codon optimised [12]. A third consideration is the site of integration, and this is determined by the plastome-derived elements that flank the transgene construct and mediate the two crossover events, as depicted in Figure 1. Several ‘neutral’ integration sites have been identified that allow integration of foreign DNA without disrupting endogenous genes or affecting the expression of nearby genes [23]. 

The final requirement for the transformation plasmid is a selectable marker. Successful delivery of DNA into the algal chloroplast is a very rare event: typically, one in 10^5^–10^6^ treated cells [11]. It is therefore essential to have a clean selection method that allows the development of colonies arising from these rare events whilst preventing the growth of any untransformed cells. Other considerations when deciding on selection strategies include: i) the likelihood of ‘false-positives’ arising through mutations that allow cells to escape from selection and give rise to colonies, and ii) the regulatory and risk issues associated with using bacterial antibiotic resistance genes as selectable markers. In this review, we focus on current selection strategies and markers developed for *C. reinhardtii* and consider the future application of markers that have been used successfully for chloroplast engineering in other algae or in higher plants. We also discuss current and future reporter genes that are invaluable in the investigation of gene expression and protein targeting within the algal chloroplast.

## 2. Selectable Markers

Selectable markers for chloroplast engineering in plants and algae can be broadly categorised into antibiotic resistance genes, herbicide resistance genes, photosynthetic genes, other positive selectable markers and negative selectable markers (Table 1). Their relative merits are considered below, as well as methods for marker removal from the transgenic plastome.

### 2.1. Selection Based on Antibiotic Resistance 

The most widely used selectable marker for chloroplast engineering is the *aadA* cassette [30]. The bacterial gene *aadA* codes for an aminoglycoside adenyltransferase (AAD), conferring resistance to spectinomycin and streptomycin: antibiotics that target the 70S ribosome of bacteria and chloroplasts. Consequently, when the coding sequence is fused to an appropriate chloroplast promoter and UTRs, *aadA* can serve as a dominant and portable marker in algae and plants (Table 1). Originally developed for *C. reinhardtii*, the marker was used initially to generate knockout mutants for the chloroplast genes *tscA, psaC* and *rpoC2* (formerly *orf472*), in order to analyse their function [30,67]. Subsequently, it has been used as a linked marker for the introduction of a GOI, as illustrated in Figure 2. Such genes include, for example, those encoding potential vaccines [16,17] or metabolic enzymes [68,69]. The *aadA* marker has also found application as a selectable marker for other microalgae that are naturally sensitive to spectinomycin, including *Euglena gracilis* [31] and *Haematococcus pluvialis* [32], and is also widely used as a selectable marker for chloroplast engineering in higher plants. First established as a marker for tobacco (*Nicotiana tabacum*) [33], *aadA* has been used in the transformation of various dicotyledonous species including potato, lettuce, soybean, cabbage, sugar beet and aubergine [70], and most recently, *Arabidopsis thaliana* [34]. The sensitivity of many dicotyledonous species to spectinomycin appears to relate, in part, to the essential nature of the plastid’s acetyl-CoA carboxylase (ACCase) enzyme, and the fact that the β-subunit of ACCase is encoded by the plastid gene *accD*. Inhibition of the translation of *accD* therefore blocks a key step in fatty acid biosynthesis, preventing tissue development [70]. In the case of *A. thaliana*, a second, nuclear-encoded ACCase is also present in the plastid and can partially compensate for the action of spectinomycin. Successful chloroplast transformation using the *aadA* marker therefore required the use of a nuclear mutant defective in this second ACCase [34]. The marker has also been used successfully to transform lower plants such *Marchantia polymorpha* [35] that possess *accD* in their plastome. However, in green algae such as *C. reinhardtii*, *accD* is not plastome-encoded so it is assumed that sensitivity to antibiotics that block chloroplast gene expression reflects some other essential aspect of the plastome [12].

A second marker for antibiotic-based selection in *C. reinhardtii* is *aphA*-6 [36]. This gene from *Acinetobacter baumannii* encodes an aminoglycoside phosphotransferase that confers resistance to kanamycin and related inhibitors of 70S ribosomes. As with the *aadA* cassette, a portable cassette for chloroplast engineering can be created by the fusion of the *aphA-6* coding sequence to suitable *cis* elements. The native coding sequence of the bacterial gene proved suitable for the chloroplast as it has a similar AT-bias to the *C. reinhardtii* plastome [36], however the Mayfield group subsequently improved the efficacy of the *aphA-6* marker through codon optimisation [71]. The marker has also been developed for use in tobacco and cotton (*Gossypium hirsutum*), although the efficiency of transformation is reported to be lower than that achieved using *aadA* [37,38].

Despite the early successes in the development of *aadA* (developed in 1991) and *aphA-6* (in 2000) as dominant antibiotic resistance markers that allow direct selection of *C. reinhardtii* transformants, there has been no further development of similar markers for this model alga. Several bacterial genes have been shown to be functional when expressed in the *C. reinhardtii* chloroplast and gave rise to a resistance phenotype, although attempts to use them for direct selection were unsuccessful. These genes include the erythromycin resistance marker, *ereB* [25] and the tetracycline resistance marker *tetX* [72]. Other bacterial genes such as *arr-2* and *cat* that confer rifampicin and chloramphenicol resistance, respectively, have been expressed in the *C. reinhardtii* plastome, but did not give rise to a resistance phenotype [72,73]. The paucity of suitable selectable markers highlights the challenges associated with their development and the various issues that need to be taken into consideration. The first involves establishing that *C. reinhardtii* is sensitive to the antibiotic within a suitable concentration range and does not give rise to numerous spontaneous resistance colonies that would complicate the recovery of bona fide transformant lines [74]. The second is choosing an antibiotic that is not light sensitive, and therefore rapidly inactivated if plates are incubated in the light. In our hands, this has proven to be an issue for both tetracycline and rifampicin [72]. The third consideration is to construct a marker that will confer sufficient resistance when present in only a few copies of the polyploid plastome, thereby allowing selection of initial heteroplasmic transformants, but is not so active that there is no selective pressure to drive selection of homoplasmic lines. This appears to be an issue with the *ereB* marker used to transform *Dunaliella tertiolecta*, as discussed below [43]. The final consideration is whether the antibiotic will affect the mitochondrion as well as the chloroplast. Both contain genetic systems that are derived from prokaryotic ancestors, albeit with a common ancestor that dates back at least two billion years. However, a few antibiotics, such as those targeting 70S-type ribosomes, might inhibit both systems. Since the mitochondrial genetic system of *C. reinhardtii* appears to be essential [75], such an antibiotic might be lethal to the cell even if local resistance is conferred within the chloroplast by expression of a selectable marker. 

A review of additional markers based on bacterial antibiotic resistance genes that have proved successful for chloroplast transformation in other species highlights those that could be developed for *C. reinhardtii*. The most promising is the *aac6-aph2* gene that encodes a bifunctional enzyme able to detoxify aminoglycoside antibiotics. This was shown to be as effective as the *aadA* marker for transformation of tobacco when used in combination with the antibiotic tobramycin [39]. Importantly, spontaneous resistance lines were not obtained with the *aac6-aph2* marker (unlike the *aadA* marker), and homoplasmy was achieved more rapidly [39]. The *aac6-aph2* gene therefore represents a potential new marker for *C. reinhardtii*, as well as other algal and plant species. A second marker that has proved successful in other species is the chloramphenicol acetyl transferase (*cat*) gene. This can be used as a selectable marker in tobacco chloroplasts, although with lower efficiency than *aadA* [40], and a codon optimised version has recently been used in the red alga *Cyanidoschyzon merolae* [41]. In the latter case, transformant lines could be readily obtained, although these could not be driven to homoplasmy, possibly due to the light-mediated breakdown of the antibiotic during prolonged selection [41]. Our own attempts to develop *cat* as a marker for *C. reinhardtii* were not successful, possibly because of a low level of expression or a failure of the protein to fold efficiently into an active form [73]. Codon optimisation might help address this, but a further concern is the possible effect of chloramphenicol on the mitochondrial translation machinery [40]. Recently, chloroplast transformation has been reported for the heterokont alga *Nannochloropsis oceanica* with the zeomycin-resistance gene, *ble*, used as the selectable marker [42]. Although *ble* is used extensively as a marker for nuclear transformation of *C. reinhardtii* and other microalgae [76], its success as a chloroplast marker is surprising. Most microalgae are highly sensitivity to zeomycin and related compounds such as bleomycin and phleomycin, which are broad-spectrum antibiotics that act by binding to DNA and inducing single- and double-stranded breaks. The resistance protein, BLE, inactivates these antibiotics not by enzymatic action, but rather by tightly binding them in a 1:1 ratio [77]. Consequently, expression of *ble* in the chloroplast would not be expected to protect the nuclear DNA from the damaging effects of zeomycin exposure unless all the drug entering the cell can be sequestered by the BLE accumulating in the organelle. A more promising marker is *ereB*, encoding erythromycin esterase. This marker has been used successfully to transform the chloroplast of the green microalga *Dunaliella tertiolecta*, although it was not possible to drive transformant lines to homoplasmy under erythromycin selection [43]. It is not clear if this failure was due to the high activity of the esterase such that there was insufficient selective pressure to increase *ereB* copy number, or whether the marker was inadvertently inserted into an essential locus, thereby preventing complete loss of the wild-type plastome [43]. Nevertheless, the success of *ereB* in *D. tertiolecta*, and our success in demonstrating functional expression (although, not direct selection) of the same *E. coli* gene in *C. reinhardtii* [25] suggests that it could be developed as an additional portable marker for *C. reinhardtii*, possibly by improving the level of expression through codon optimisation. Finally, a second kanamycin resistance gene has been developed for plant chloroplasts. The neomycin phosphotransferase gene, *nptII*, works as a selectable marker in tobacco, albeit with lower efficiency than the *aphA-6* marker [37,44]. In cotton, a higher transformation efficiency is achieved on kanamycin-containing selective media when *nptII* is used in combination with *aphA-6* rather than using *aphA-6* alone [38]. Whilst *aphA-6* already works well as a kanamycin resistance marker in *C. reinhardtii*, the strategy of employing two different enzymes that target the same antibiotic or using a bifunctional enzyme such as AAC(6′)-APH(2′′) that inactivates an antibiotic through two different modifications [39] might prove useful in the development of new and more efficient selection methods.

**Figure 3:**
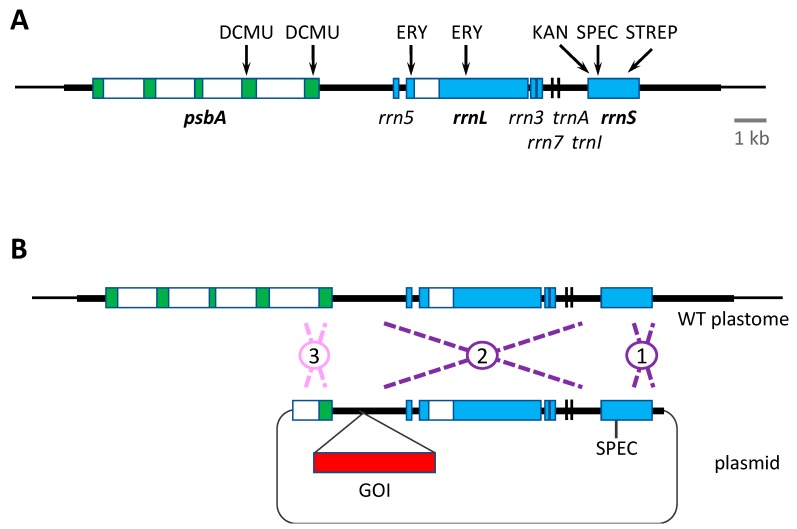
Dominant mutations in *C. reinhardtii* chloroplast genes that can be used for selection. (**A**) The gene organization within the inverted repeat region (thick black line) of the plastome with the location of point mutations in *psbA* giving rise to herbicide resistance (DCMU), those in *rrnL* conferring erythromycin resistance (ERY), and those in *rrnS* conferring resistance to kanamycin (KAN), spectinomycin (SPEC) and streptomycin (STREP) [45,47]. Introns in *psbA* and *rrnL* are indicated as white boxes. (**B**) The constraints of using such markers. Targeting a GOI to a neutral site such as downstream of *psbA* using SPEC, for example, involves constructing a large plasmid in which the GOI and SPEC are separated by many kilobases. In this situation, integration via a double crossover is much more likely to occur as events 1 + 2 rather than 1 + 3, resulting in a preponderance of ‘marker-only’ transformants. A separation of only a few hundred bases can still give rise to a significant percentage of such transformants [12]. WT = wild-type.

All the above antibiotic resistance cassettes represent dominant markers that are portable. That is, they can be used to transform the wild-type plastome and can be targeted to any desirable locus by simply flanking the marker (and any additional GOI, as shown in Figure 2) with appropriate plastome sequences. However, the downsides of such markers are: (i) the presence of an undesirable antibiotic resistance gene in the resulting genetically modified organism (GMO), and thus the risk of horizontal gene transfer to other microorganisms and the additional regulatory considerations for exploitation of such GMOs; (ii) the unnecessary metabolic burden of expressing a no-longer required marker gene, together with the possibility of unwanted competition for specific *trans*-acting factors between *cis* elements common to the marker gene, endogenous chloroplast genes and/or the introduced GOIs. An alternative antibiotic-based selection involves the use of variants of the chloroplast *rrnS* and *rrnL* genes encoding the 16S and 23S ribosomal RNAs, respectively. Specific point mutations in *rrnS* can give rise to ribosomes resistant to spectinomycin, streptomycin or kanamycin, whereas mutations in *rrnL* can give rise to erythromycin resistance (Figure 3A). These variants can therefore be used as endogenous markers such that homologous recombination replaces the sensitive (i.e., wild-type) allele in the plastome with the antibiotic resistant allele [45]. However, the drawback is that these markers are not portable, and consequently any transgene to be introduced needs to be targeted into a neutral site within the inverted repeat region where the *rrnS* and *rrnL* genes are located. This can result in a transformation construct in which there is a significant distance between the antibiotic resistance determinant and the GOI. Consequently, there is the potential for recombination in the intervening region, resulting in integration of the selected *rrn* allele but not the GOI. The greater the distance, the higher the risk of ‘marker-only’ transformant lines as shown in Figure 3B. An alternative strategy is to completely decouple the marker and the GOI by co-transformation with two plasmids: one carrying the *rrn* allele and a second carrying the GOI flanked by elements for recombination at a desired locus [74]. Again, this approach can result in a significant percentage of marker-only transformants, but does allow the targeting of the GOI to any locus. A final consideration when using the *rrn* alleles as markers is the negative impact of the antibiotic resistance mutation on ribosome performance. Equivalent mutations in bacteria are often associated with reduced fitness [78], and if the same is true in the chloroplast, then the maximum yield of recombinant protein, especially for highly expressed transgenes, might be compromised in transgenic lines carrying such mutations.

### 2.2. Selection Based on Herbicide Resistance

Similar to the point mutations in the ribosomal RNA genes conferring antibiotic resistance, missense mutations in the chloroplast *psbA* gene encoding the D1 subunit of photosystem II (PSII) have been identified that confer resistance to herbicides such as DCMU, metribuzin and phenmedipham [46,47]. As with the *rrn* mutants, these *psbA* variants can be used as dominant endogenous markers, although they suffer from the same issues as discussed above and illustrated in Figure 3, of not being portable and possibly impacting PSII efficiency in the transgenic lines. 

To date, no portable markers conferring herbicide resistance have been developed for the *C. reinhardtii* chloroplast, although the *bar* gene is a promising candidate. The gene encodes a phosphinothricin acetyltransferase (PAT) that inactivates the herbicide phosphinothricin—an inhibitor of glutamine synthetase (GS) [79]—and has been employed as a marker for chloroplast transformation of tobacco and the green alga *Platymonas subcordiformis* [48,49]. In the tobacco study, both bacterial and codon optimised versions of *bar* were shown to yield phosphinothricin-resistant plants, however, direct selection of chloroplast transformants on the herbicide could not be achieved. This is possibly due to the initially low level of PAT in only a few chloroplasts within the transformed plant cell, and the fact that it is the cytosolic isoform of GS (rather than the chloroplast form) that is more active in leaf tissue. Inhibition of GS by phosphinothricin results in a cytotoxic build-up of ammonia, and consequently, low-level PAT activity in the chloroplast may be insufficient to detoxify all the herbicide and therefore prevent the lethal inhibition of the GS in the cytosol and in untransformed chloroplasts [48]. In contrast, Cui et al. reported direct selection on phosphinothricin for chloroplast transformants of the green alga *P. subcordiformis* using the *bar* gene fused to regulatory chloroplast elements derived from *C. reinhardtii* [49]. This success possibly reflects the fact that *P. subcordiformis* possesses only a single large chloroplast, unlike higher plant cells, and therefore the initial expression of *bar* is sufficient to confer ‘whole cell’ protection to phosphinothricin [49,80]. This result bodes well for using the same *bar* cassette as a dominant, portable marker for *C. reinhardtii*. However, selection should be carried out using methionine sulfoximine—a structurally related GS inhibitor that is also detoxified by PAT [81]—as wild-type *C. reinhardtii* is resistant to phosphinothricin but highly sensitive to methionine sulfoximine [82].

Several other herbicide-resistant or herbicide-insensitive genes have been explored as chloroplast markers in plants and might have application for *C. reinhardtii*. In most cases, the transformed plants showed a tolerance to the herbicide, but direct selection on the herbicide for primary transformants was not successful (e.g., [83]). This is possibly for the same reason as discussed above for the *bar* gene, that only a sub-population of the multiple chloroplasts within the cell of a primary transformant have acquired the transgene, and therefore there is insufficient protection of the whole cell or developing tissue at this stage [80]. This is particularly an issue when employing herbicide-insensitive variants of endogenous chloroplast enzymes as markers, rather than novel enzymes that metabolise the herbicide to a non-toxic product. An example of this is a mutant form of the bacterial *hemL* gene that encodes a gabaculine-insensitive variant of the enzyme glutamate 1-semialdehyde aminotransferase (GSA) [50]. In photosynthetic eukaryotes, GSA is localised in the chloroplast and plays a central role in chlorophyll synthesis. Gabaculine-mediated inhibition of GSA results in chlorosis and cell death in plants and algae, including *C. reinhardtii* [84]. When the *hemL* gene was over-expressed in the tobacco chloroplast, the plants became insensitive to gabaculine [50], but attempts to use *hemL* for direct selection were unsuccessful. Other possible markers include bacterial genes encoding 5-enolpyruvylshikimate-3-phosphate synthase (EPSPS), a key enzyme involved in the biosynthesis of aromatic amino acids. Unlike the plastidic forms of EPSPS, the bacterial enzymes are tolerant to high levels of the competitive inhibitor, glyphosate, and could therefore be exploited in *C. reinhardtii* as a selectable marker [85]. Alternatively, the *Bacillus* gene encoding glyphosate acetyltransferase could be explored as a detoxifying marker for the algal chloroplast [86]. Finally, a mutant form of the *ahaS* gene from the red alga *Porphyridium* sp. has potential as a dominant marker for *C. reinhardtii*. The gene encodes acetohydroxyacid synthase (AHAS), a chloroplast-localised enzyme that catalyses the first step in the synthesis of branched-chain amino acids, and is the target of sulfonylurea herbicides such as sulfometuron methyl (SMM). In higher plants and green algae the AHAS gene is located in the nucleus, whereas in *Porphyridium,* the gene is found in the chloroplast genome. Importantly, a mis-sense mutation in the *Porphyridium* gene (*ahaS*^W492S^) results in resistance to SMM, and this mutant gene has been reported to function as a dominant marker for chloroplast transformation of the red alga [51].

### 2.3. Selection Based on Restoration of Photosynthesis

**Figure 4:**
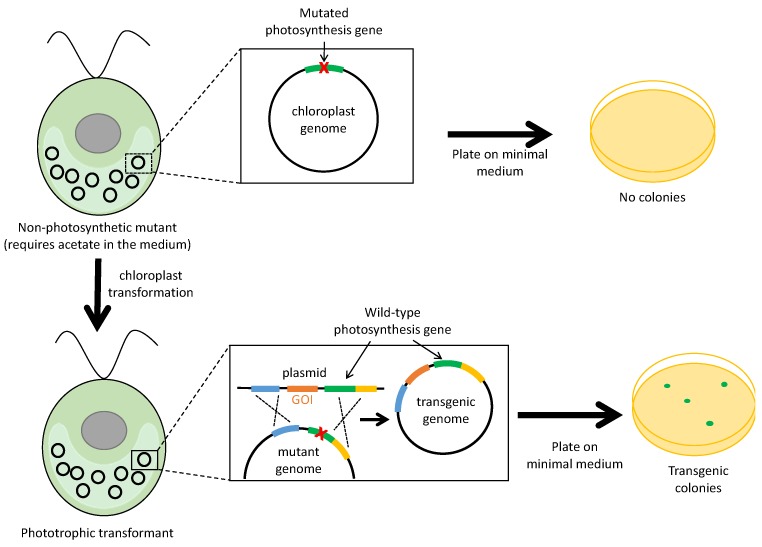
Selection based on photosynthetic rescue. The recipient *C. reinhardtii* strain is incapable of phototrophic growth owing to a deletion or point mutation in an essential photosynthesis gene and must be maintained on acetate-containing medium. Transformation of the recipient with donor DNA including the wild-type version of the gene results in restoration of photosynthesis and concomitant integration of the GOI, enabling transformants to grow on a minimal medium.

Whilst the various selection methods described above can be used to transform wild-type strains of *C. reinhardtii*, an alternative strategy is to exploit the fact that this species can dispense completely with photosynthesis and grow heterotrophically on a carbon source such as acetate. Consequently, many photosynthetic mutants have been described in the literature, including those carrying deletions or point mutations in the plastome that disrupt essential photosynthesis genes [4]. Transformation of such strains with a wild-type copy of the affected gene provides a powerful selection since homologous recombination restores a functional copy of the gene to the plastome, allowing selection for a wild-type phenotype on minimal medium (i.e., lacking acetate). As a consequence, homoplasmy is achieved more readily than with antibiotic-based markers such as *aadA* and homoplasmic lines can been obtained even after a single round of single-colony isolation [25]. This reduces the time to generate stable transformants to 3–4 weeks [74]. Furthermore, the use of a deletion mutant as the recipient strain avoids the appearance of any ‘false-positive’ colonies as it is not possible for the strain to spontaneously revert to phototrophy. However, perhaps the most appealing aspect of using an endogenous gene for selection is that transformant lines are “marker-free” [52,53]. As illustrated in Figure 4, a GOI can be targeted to a neutral site upstream or downstream of the photosynthetic gene by incorporating it into one of the flanking elements within the transformation construct. The resulting transformant contains only the GOI as foreign DNA in its plastome, thereby alleviating concerns of escape and horizontal transfer of antibiotic resistance markers such as *aadA*. Importantly, the use of the wild-type allele of an endogenous gene as a selectable marker does not risk compromising translational or photosynthetic efficiency, unlike the markers based on dominant point mutations in the *rrnS/L* genes or *psbA*, respectively, as discussed in Section 2.2.

Non-photosynthetic chloroplast mutants of *C. reinhardtii* that have been employed as recipients for such phototrophic rescue include those carrying large deletions affecting *tscA*, *psbA* and *atpB* [30,53,54]. Indeed, the first demonstration of stable chloroplast transformation involved the rescue of an *atpB* deletion mutant to prototrophy by biolistic transformation with the cloned *atpB* gene [54]. Other mutants that can also be used as recipients include those with frameshifts in *atpE* [55] or *psaB* [56], or a nonsense mutation in *rbcL* [52]. However, these are less suitable than the deletion mutants because of the likelihood of ‘false-positive’ revertants, or possible recombination either side of the point mutation resulting in restoration of the wild-type sequence without integration of the linked GOI. An alternative strategy to using existing chloroplast mutants recovered from early forward-genetic screens is to create new knockout mutants by chloroplast transformation in which an essential photosynthesis gene is deleted using the *aadA* cassette. These transgenic strains can then be used for subsequent transformation in which the wild-type gene is used for selection, replacing *aadA* and restoring phototrophy. The advantages here are that the extent of the deletion can be precisely controlled when creating the recipient, and the loss of *aadA* (and therefore loss of spectinomycin resistance) serves as a useful phenotypic test for homoplasmy. Two examples of such engineered recipients are a *petB*::*aadA* strain [57] and a *psbH*::*aadA* strain [25]. 

Finally, a variation on the strategy of using an endogenous gene as a marker to rescue a non-photosynthetic strain was described recently [58]. Here, the recipient strain carries a nonsense mutation in the photosystem I (PSI) gene, *psaA-3,* that changes a tryptophan codon to the UGA stop codon. Rescue of the strain to phototrophy was achieved by transformation with a construct carrying a modified version of the chloroplast gene *trnW* that encodes the tryptophan tRNA. A single base change in the anticodon allows the modified tRNA to recognise UGA as a tryptophan codon and therefore translate *psaA-3* to yield the full-length PSI subunit. This is particularly elegant as the *trnW* marker is small (at 275 bp, it is by far the smallest chloroplast marker yet described) and portable since the tRNA acts *in trans* to suppress the mutation in *psaA-3*. The marker can therefore be tightly linked to a GOI and introduced anywhere in the plastome.

### 2.4. Other Positive Selection Markers

Whilst the commonly-used *C. reinhardtii* selectable markers fall into the categories discussed above, a few selection systems for chloroplast transformation have been described that do not fall into these categories but are worth noting.

The ability of wild-type *C. reinhardtii* to synthesise arginine is reliant on a biosynthetic pathway that is predominantly located within the chloroplast. A key enzyme in this pathway is *N*-acetyl ornithine aminotransferase, encoded by the nuclear gene, *ARG9*, and mutants affected in this gene require arginine-containing media to survive [59]. The *ARG9* cDNA from *Arabidopsis thaliana* was shown to function as a selectable marker for chloroplast transformation of such mutants, restoring the ability to synthesise arginine and thus enabling selection on media lacking arginine [59]. *ARG9* therefore represents a useful portable marker and, as a foreign gene encoding an enzyme of basic metabolism rather than an antibiotic resistance enzyme, it is preferable when generating strains for industrial applications since the ability to synthesise arginine does not carry the same risks associated with horizontal transfer. Another metabolic enzyme that has recently been developed as a selectable marker for the *C. reinhardtii* chloroplast is the bacterial *ptxD* gene encoding phosphite oxidoreductase [60]. Eukaryotes and most prokaryotes are unable to utilise phosphite (PO_3_^3−^) as a source of phosphorus, however the PtxD enzyme oxidises phosphite to the bioavailable form, phosphate (PO_4_^3−^). Consequently, *ptxD* can be used as a general marker for transforming wild-type microorganisms including *C. reinhardtii* by selecting for growth on medium containing phosphite as the sole source of phosphorus [60,87].

Several other metabolic markers suitable for plant chloroplast transformation have been described in the literature and could have application in *C. reinhardtii*. For example, a variant of the tobacco *ASA2* gene encoding the α-subunit of anthranilate synthase has been shown to allow direct selection for tobacco transformants on the tryptophan analogues 7-methyl-DL-tryptophan and 4-methylindole [61]. Anthranilate synthase is the first enzyme in the tryptophan biosynthesis pathway and is regulated by tryptophan via a negative feedback mechanism. Tryptophan analogues are therefore toxic to wild-type plants as they mimic tryptophan, thereby downregulating anthranilate synthase activity and causing tryptophan deficiency. However, the ASA2 variant is insensitive to feedback regulation and therefore plants expressing the gene can be selected using the analogues [61]. This system could have application in *C. reinhardtii*, which shares the same tryptophan biosynthesis pathway [88], provided that a suitable *ASA2* variant could be identified, or that the tobacco gene could be successfully expressed in the algal chloroplast. Other selection strategies that are less likely to be transferrable to *C. reinhardtii* are those based on the yeast (*dao*) or bacterial (*dsdA*) genes that confer resistance in tobacco by metabolising the toxic amino acids, D-alanine and D-serine, respectively [62,63]. Early work on uptake of exogenous amino acids by *C. reinhardtii* have shown that arginine is the only amino acid actively transported into the cell [88]. Furthermore, D-alanine has been shown to have no inhibitory effect on the growth of the alga [89]. Finally, an early study reported that the spinach *BADH* gene encoding betaine aldehyde dehydrogenase could serve as a highly efficient marker for the direct selection of tobacco chloroplast transformants [64]. The selection process involved the conversion of toxic betaine aldehyde by BADH to non-toxic glycine betaine, and was particularly attractive as it involves a gene from an edible plant as the marker rather than an antibiotic-based marker such as *aadA* [64]. However, these results have proved difficult to repeat [90,91], and the marker might not be suitable for *C. reinhardtii* as the alga already contains an endogenous BADH enzyme [92]. 

### 2.5. Negative Selectable Markers

**Figure 5:**
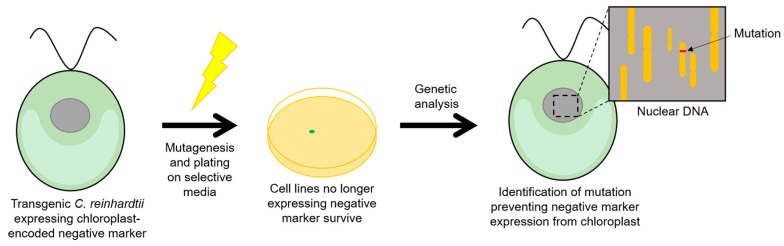
Use of negative selectable markers to identify nuclear-encoded factors required for chloroplast gene expression. A transgenic line is established expressing the negative marker (e.g., *crCD* [66]) in the chloroplast under the control of an endogenous promoter/5′ UTR element. Subsequently, a forward-genetic screen is performed by random mutagenesis to recover colonies able to survive on the selective media, including those mutants no longer expressing both the negative marker and the endogenous gene because of the loss of a nuclear-encoded factor. Phenotypic and western analysis is used to confirm such mutants, and genetic analysis identifies the nuclear gene encoding the factor [66].

Negative selectable markers confer a deleterious phenotype to transformants, and whilst unsuitable for direct selection, such markers can be useful tools in the study of gene regulation and as counterselection systems for the removal of positive selectable markers or other undesired DNA sequences. An example of a conditional negative marker is the *E. coli* cytosine deaminase gene, *codA* which confers sensitivity to 5-fluorocytosine (5-FC), converting it to the cytotoxic product 5-fluorouracil. This gene has been shown to function in tobacco chloroplasts, giving rise to plants that are highly sensitive to 5-FC and allowing seedling-based screens for mutant lines that fail to express *codA* [65]. More recently, a codon optimised and modified version of *codA* (termed *crCD*) was expressed in the *C. reinhardtii* chloroplast and exploited for similar forward-genetic screens [66]. The *crCD* marker contains changes to specific residues that increase the affinity of the cytosine deaminase for 5-fluorocytosine, and was used in a screen to isolate mutants of the nuclear-encoded factors MCA1 and TCA1 that are known to be required for translation of the chloroplast *petA* transcript through interaction with the 5′ UTR. Chloroplast transformants containing *crCD* under the control of the *petA* promoter/5′ UTR were sensitive to 5-FC, but, following mutagenesis, resistance mutants were recovered as illustrated in Figure 5 and shown to carry mutations in either *MCA1* or *TCA1* [66]. This demonstrates the utility of such negative selectable markers for targeted screens aimed at identifying factors required for protein expression in the chloroplast and aiding our understanding of chloroplast gene expression. In turn, this may be useful in optimising systems for producing valuable biomolecules; for example, by engineering the over-expression of a required factor in order to increase the expression of a GOI. 

The other negative marker that has been developed for chloroplast engineering is the yeast *dao* gene that confers sensitivity to D-valine [62]. Whilst this marker is unlikely to be suitable for *C. reinhardtii* because of the limited uptake of D-valine into the cell, as discussed in the previous section, other conditional negative selection strategies could be explored based on those used in the plant nucleus [93]. Negative markers are useful not just for targeted loss-of-function screens, but also as molecular tools for driving the loss of DNA from the chloroplast genome through recombination between direct repeats. This can be employed to eliminate unwanted positive selectable markers following recovery of transgenic lines as discussed in the next section.

### 2.6. Removal of Selectable Markers

As discussed above, a range of different selection strategies have been developed for both algae and plants. However, the most widely-used system remains the *aadA* marker conferring spectinomycin resistance [24]. Given that the plastome of algae and plants is highly polyploid (typically 80 in a *C. reinhardtii* cell [6], but as many as 2000 in a plant mesophyll cell [24]), the presence of so many copies of *aadA* per cell raises a significant concern when considering commercial applications. Spillage of the transgenic DNA into the environment or into an animal gut would allow uptake by microorganisms including bacterial pathogens. This obviously presents regulatory and risk barriers to exploitation of transgenic lines containing *aadA* or other antibiotic resistance markers. Consequently, methods have been developed for both *C. reinhardtii* and plants that circumvent such problems by enabling the removal of the marker gene after transformants have been identified. An additional advantage of such systems is that they allow the marker gene to be re-used, allowing successive rounds of transformation and selection to generate transgenic cells expressing multiple GOIs.

**Figure 6:**
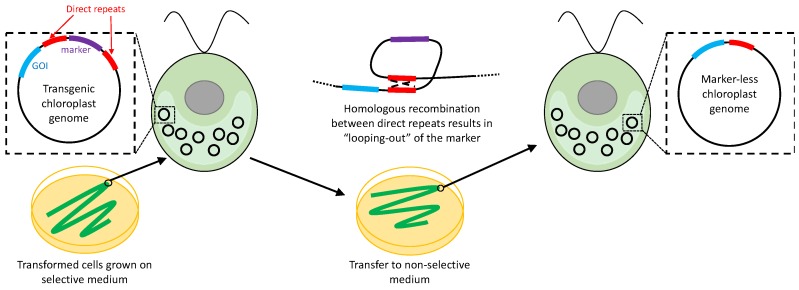
Marker removal based on direct repeats. Once the selective pressure to keep the selectable marker is removed, homologous recombination occurs between repeats flanking the marker, resulting in loss of the marker from the chloroplast genome [94].

Two separate strategies were developed by Fischer et al. [94] for the removal of *aadA* from the *C. reinhardtii* plastome, both based on loss of the marker gene during strain propagation once the selective pressure for survival on spectinomycin has been removed. The first, and arguably simpler, approach involves flanking both sides of the marker gene with a long direct repeat, with a GOI placed outside the repeats. Transformants are first plated on the selective medium as usual, and re-streaked to single colonies on this medium until homoplasmy has been established. The transformants are then propagated on non-selective medium to allow loss of the marker. Providing that the flanking direct repeats are sufficiently long, excision of the marker occurs naturally, presumably by a ‘looping out’ mechanism involving homologous recombination between the repeats as shown in Figure 6. Similar strategies have subsequently been used in higher plants to create marker-less lines with GOIs inserted into the plastome [95], or with endogenous genes deleted from the plastome [96]. 

However, there are technical drawbacks with this strategy. Firstly, the efficiency of ‘looping out’ appears to be dependent on the length of the repeat. For *C. reinhardtii*, 90% of *aadA* transformants lost spectinomycin resistance when an 832 bp flanking repeat was used; this decreased to 40% if the repeat was 483 bp, and 0% at 230 bp [94]. In tobacco, shorter repeats (174 bp) are still effective [95], but nonetheless the final transgenic line in all cases retains a single copy of the repeat within the plastome, as shown in Figure 6. Consequently, re-use of the same repeat element for GOI insertion elsewhere in the plastome could then create plastome instability through recombination between these copies. A further problem is that there is no active selection for loss of the marker, with the recovery of marker-less *C. reinhardtii* lines dependent just on random partitioning and spread of marker-less plastome copies in the progeny following plating on the non-selective medium. This therefore necessitates repeated re-streaking and laborious screening of putative lines for the loss of the marker [94]. One strategy to overcome this would be to include a negative selectable marker such as *crCD* [66] together with *aadA* between the repeat elements. This would allow positive selection for loss of the *aadA*-*CrCD* dual marker based on acquired resistance to 5-FC. A similar strategy for plant chloroplasts using *aadA* together with the *dao* marker, and selection for resistance to D-valine, has been proposed by Gisby et al. [62]. 

The second approach taken by Fisher et al. [94] for marker elimination in the *C. reinhardtii* chloroplast involves co-transformation with two plasmids: one containing the *aadA* marker cloned within the coding region of an essential chloroplast gene, and the other containing the GOI flanked by a chloroplast sequence that would target it to a second, neutral locus. Selection on spectinomycin generates *aadA* transformants, with some lines also containing plastomes transformed with the second plasmid. However, homoplasmy cannot be achieved for the gene knockout as the cells are unable to survive without some functional copies of the essential gene, and a persistent heteroplasmy is observed when lines are maintained on spectinomycin. At the same time, the GOI insertion can reach homoplasmy amongst the plastome population, which can be checked by PCR. Cell lines in which this homoplasmic state is confirmed are then transferred to a non-selective medium, and the plastome copies carrying the selectable marker gene are rapidly lost due to selective pressure to restore full function of the essential gene. The advantage of this method is that the resulting transformant lines contain only the GOI without any additional sequences, such as a copy of a direct repeat. The downside is that there is no selection for the integration and homoplasmy of the GOI, so identifying such lines can be time-consuming and laborious, requiring multiple rounds of growth on both selective and non-selective media [94]. 

**Figure 7:**
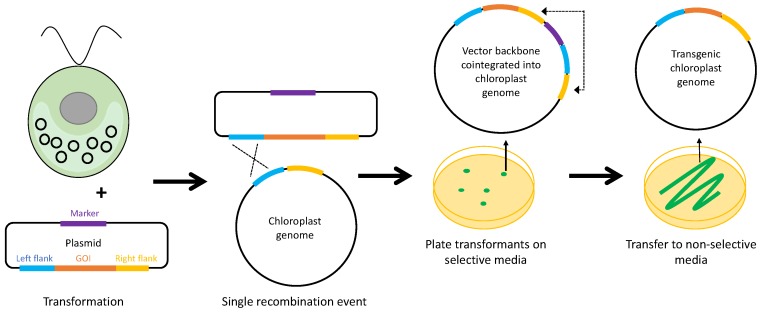
Co-integration of a selectable marker. The marker is placed outside of the flanking regions in the transformation plasmid, within the vector region. Upon transformation, an initial, single recombination event results in co-integration of the whole plasmid, including the marker, into the plastome. Antibiotic selection for the marker allows the maintenance of the unstable co-integrated state, but, once the selective pressure is removed, a second recombination between a pair of homologous elements is favoured, resulting in one of two possible outcomes: recombination between the original elements (in blue) and loss of the whole plasmid restoring the wild-type plastome, or recombination between the second elements (in yellow) to create a transgenic line containing just the GOI. The latter is identified by PCR [97].

Alternative systems for marker removal have been described for plant chloroplasts, and could be applied to *C. reinhardtii*. One interesting selection system has been proposed that employs a ‘built-in’ marker removal system [97]. As illustrated in Figure 7, this system relies on the theory that, when the chloroplast is transformed with a plasmid containing two DNA elements with homology to the plastome, there is an initial recombination event with one of these elements which leads to the co-integration of the entire vector backbone. Another recombination event with the other element (or a reverse of the initial recombination) will follow, as the plastome carrying the co-integrate contains two pairs of direct repeats, making this inherently unstable. However, by including the selectable marker outside the elements in the plasmid vector rather than inside (as in Figure 2), the co-integrated vector is maintained in the plastome and can be driven to homoplasmy as long as selection is applied. Once the selection is removed, the plasmid backbone portion containing the marker is rapidly lost through recombination between one of the two pairs of repeat elements, resulting in either a marker-less plastome carrying the GOI or restoration of the original untransformed plastome [97].

An alternative strategy for marker removal from the plastome involves the use of bacteriophage site-specific recombinases rather than the chloroplast’s endogenous recombination machinery to excise the marker DNA [98]. This approach is now well-established for engineering of plant chloroplasts, but the method has been demonstrated only for marker excision from the *C. reinhardtii* nucleus [99] and has yet to be demonstrated for the chloroplast. Various recombinases have been shown to work in the tobacco chloroplast, including Cre recombinase [100], phiC31 [101] and Bxb1 [102]. These systems all work similarly and employ a two-step approach: a selectable marker flanked by recognition sequences specific to the recombinase is used for chloroplast transformation, and then, after selection of transplastomic lines, a gene encoding a chloroplast-targeted version of the recombinase is introduced into the plant nucleus. This latter step can be achieved either by direct nuclear transformation of the transplastomic line, or by crossing the transplastomic line with a second line known to express the recombinase transgene [99]. The recombinase localises to the chloroplast and excises the DNA between the recognition sequences resulting in loss of the marker from the plastome.

All three recombinase systems show a high efficiency in removing the marker and could readily be applied to *C. reinhardtii* since both sexual crosses and nuclear transformation are well-established for this species [4,7]. However, a drawback of the strategy is the requirement for a nuclear-encoded recombinase. This adds additional experimental steps to the process of generating a marker-less transformant, and also introduces an additional consideration given that engineering of the nuclear genome might itself involve the use of an antibiotic resistance marker. This nuclear marker would need to be removed from the final transformant line by further rounds of back-crossing to a wild-type strain. Another issue observed in the tobacco chloroplast when using the Cre recombinase is that this enzyme seems to mediate recombination between cryptic *loxP* sequences within the plastome in addition to the bona fide target sequence, and also appears to stimulate homologous recombination between endogenous non-*loxP* sequences that are as short as 117 bp [103]. The application of this technology to the *C. reinhardtii* chloroplast therefore requires consideration of recombinases for which the plastome lacks possible cryptic target sites, and the use of recessive endogenous markers such as *ARG7* for the nuclear engineering step [7]. 

## 3. Reporters

Scorable reporter genes can prove useful in both the improvement of genetic engineering protocols and in the study of native biological processes. These genes encode proteins that confer an easily detectable phenotype on the transformant, often allowing for rapid and simple screening procedures or assays that do not necessitate laborious diagnostic tests to confirm and quantitate expression levels [104]. Reporter genes are therefore important tools in the advancement of *C. reinhardtii* chloroplast engineering, and those available are discussed below. 

### 3.1. Resistance Markers or Endogenous Genes as Reporters

As antibiotic or herbicide resistance genes confer an easily scorable phenotype (i.e., resistance to a certain concentration of the drug), it is possible to use such markers as reporters. For example, the *aadA* gene [30] has been fused to the 5′ UTR of different chloroplast genes, e.g., *psbC* and *psaA,* to demonstrate that specific nuclear-encoded factors are required for translation of that gene, and in the absence of the factor, no spectinomycin resistance is observed [105,106]. The *aadA* protein (AAD) can also be used to probe the topology of thylakoid membrane proteins by creating AAD fusions to different domains of a membrane protein [107]. Here, high spectinomycin resistance is observed if AAD localises to the stromal side of the thylakoid membrane, whereas low resistance is seen if AAD is within the thylakoid lumen [107]. 

A colour phenotype also makes for an attractively simple assay. In green algae, three chloroplast genes (*chlB*, *chlL* and *chlN*) encode components of an enzyme involved in a key step of chlorophyll synthesis. Importantly, this enzyme does not require light as a substrate, unlike a second nuclear-encoded enzyme that carries out the same step in the chloroplast in a light-dependent manner [108]. Consequently, colonies of chloroplast mutants defective in one of the *chl* genes are viable but have a ‘yellow-in-the-dark, green-in-the-light’ phenotype. This could be exploited as a simple binary reporter system in which a wild-type copy of the *chl* gene (e.g., under the control of a range of different synthetic promoters) is introduced into the mutant plastome, and expression assayed by scoring dark-grown colonies for a switch from yellow to green [57].

### 3.2. β-glucuronidase as a Reporter

The *E. coli* gene *uidA,* which encodes β-glucuronidase (GUS), is a widely used reporter for higher plant and algal studies since these organisms lack endogenous GUS activity [109]. Furthermore, GUS is a highly active and stable enzyme that catalyses the hydrolysis of a wide range of artificial β-glucuronide compounds. As such, histochemical assays of whole cells are feasible when using X-gluc as the substrate to produce a blue product, and quantification of GUS activity in cell extracts is possible using fluorometric or spectrophotometric assays [110]. The reporter can therefore be used to accurately assess the activity and strength of different promoter and UTR elements. Furthermore, the GUS protein is also tolerant of both N- and C-terminus fusions, allowing studies of the localisation and accumulation of proteins in cells [110]. The first reported example of GUS use in *C. reinhardtii* chloroplasts was in the study of the 5′ UTR of the *petD* gene, illustrating that this region is essential for the stability and translation of the *petD* mRNA [111]. In separate studies, GUS activity was used to assess the promoters and 5′ UTRs from three different chloroplast genes, *rbcL*, *psbA* and *atpA* [112], and to identify important sequence determinants for translation [113]. The work identified the *atpA* regulatory regions as those giving the highest expression levels and highlighted the importance of sequences immediately upstream of the start codon, illustrating the value of such reporters in informing the choice and design of the 5′ UTR to achieve the maximum yield of a heterologous protein. However, a disadvantage of using GUS is that it is not a vital reporter—i.e., measurements cannot be made in living cells, as the cells are killed during the assay process [110]. Consequently, the GUS reporter is not suitable for certain experiments, such as temporal studies of changing protein levels within a single cell, or forward-genetic screens of cell populations. 

### 3.3. Fluorescence-Based Reporters

In contrast to GUS, fluorescent proteins (FPs), such as green fluorescent protein (GFP) from the jellyfish *Aequorea victoria,* can serve as vital reporters. They are stable and benign proteins that can accumulate in living cells to levels where their inherent fluorescence following excitation with shorter wavelength light is readily detectable by fluorescence microscopy or fluorimetry [114]. However, despite the widespread application of FPs as reporters for cell biology studies, attempts to exploit these reporters for studies of the *C. reinhardtii* chloroplast have so far been disappointing. Early work by Franklin et al. [28] showed that codon optimisation and introduction of the well-characterised S65T mutation [114] into the original GFP gene improved expression levels (to 0.5% of total soluble protein) and allowed fluorescence detection in cell extracts using a microplate assay. However, attempts to detect GFP in whole cells by fluorescence microscopy were not successful [28]. At the same time, Kobe et al. [115] reported the expression of a GFP variant in the *C. reinhardtii* chloroplast using a synthetic gene codon optimised for expression in the tobacco chloroplast. This variant carried S65G and S72A changes to the protein, which result in a red-shift of the excitation maximum, and accumulated to 5% of total leaf protein in transplastomic tobacco, allowing fluorescence imaging of chloroplasts in plant tissue [116]. In the *C. reinhardtii* chloroplast, the level of GFP accumulation was approximately 100-fold less, although just sufficient to allow fluorescence detection of the chloroplast in whole cells [115]. In order for FPs to be useful as tools for *C. reinhardtii* studies in vivo, it is clear that accumulation levels in the chloroplast need to be improved. The need for a high abundance of an FP is in part due to the high concentration of chlorophylls and other fluorescent molecules present in the cell that absorb some of the light used to excite the FP resulting in strong auto-fluorescence ‘noise’ that can obscure the FP emission [117].

A few attempts to address these problems by using FPs with different spectral properties to GFP have been met with limited success. The vivid verde fluorescent protein (VFP), a FP related to GFP from the coral *Cyphastrea microphthalma,* was codon optimised and successfully expressed in the *C. reinhardtii* chloroplast, but fluorescence levels were low and could only be detected in whole cells by flow cytometry [118]. Similarly, a cyan FP (mTurquoise2) derived from GFP that has been developed as a chloroplast reporter for the liverwort *Marchantia polymorpha* [119] was codon optimised for *C. reinhardtii* and shown to give a detectable signal in fluorimetry assays of transformant cells [120]. However, neither VFP nor mTurquoise2 transformants had fluorescence levels sufficient to allow microscope imaging or colony screens [118,120]. On the positive side, studies of nuclear transformants of *C. reinhardtii* expressing a range of FPs indicate that those such as tdTomato and mVenus that have red-shifted excitation and emission maxima relative to GFP are clearly superior in terms of good signal-to-noise ratios as auto-fluorescence from the cell is relatively low at these maxima [117,121]. These two FPs therefore represent good candidates for future development of chloroplast reporters.

### 3.4. Luciferase-Based Reporters

The luciferase enzymes represent a second class of vital reporter that can be used for in vivo studies. Unlike fluorescent proteins, luciferases are based on bioluminescence—i.e., these enzymes catalyse the emission of photons of light from a substrate without requiring exogenous illumination [122]. Although bioluminescence signals are weaker than fluorescence, luciferase systems often offer more sensitive detection than FPs owing to the very low background levels of bioluminescence in most cells. A second advantage is that exogenous illumination is not required, simplifying the assays and avoiding bleaching of the reporter protein, or phototoxic damage to the cell [122]. Three different luciferase systems have been developed for the *C. reinhardtii* chloroplast based on the genes from the soft coral *Renilla reniformis* [123], the bacterium *Vibrio harveyi* [124] and the firefly, *Photinus pyralis* [125], as detailed in Table 2. 

Various reports illustrate the value of these luciferases for investigating the intricacies of gene expression in the *C. reinhardtii* chloroplast. All three reporters allow detection and quantification of the bioluminescent signal in living cells or colonies, and have been used to follow temporal changes in gene expression, or identify factors required for expression of individual chloroplast genes. For example, *lucCP* was used to monitor chloroplast circadian rhythms, demonstrating that the circadian period is under nuclear control [125], and has also enabled the identification of a nuclear factor necessary for expression of the photosystem I gene, *psaC* [126]. The bacterial luciferase, *luxCt*, has been used to illustrate the light-activated translation of the photosystem II gene, *psbA* [124] and to test different promoter/5′ UTR combinations for high levels of transgene expression [27]. In addition, when *luxCt* was fused to the *rbcL* gene encoding a subunit of rubisco, the most abundant protein in the chloroplast, a very high luminescence and abundance of LuxCt was observed, highlighting a potential strategy to increase heterologous protein expression in the *C. reinhardtii* chloroplast [71]. Further improvements to the luciferase reporters could allow more sophisticated assays. For example, codon optimisation of *rluc* would be expected to improve its expression, and therefore improve its utility given that it is the smallest of the three and requires no co-factor (Table 2). The *rluc* gene could also be used in combination with *lucCP* given that they have different light emission maxima, in order to follow several different gene expression events in the same chloroplast. Furthermore, the ever-expanding list of luciferases isolated from different organisms [127] offers other potential reporters for the chloroplast, such as those enzymes with emission maxima in the green region of the spectrum that would avoid excessive quenching by light-harvesting pigments. Finally, some of the smaller luciferases are particularly attractive as tools for protein tagging studies and include the 19 kDa G-Luc from marine copepod *Gaussia princeps,* which has already been used successfully as a reporter of nuclear gene expression in *C. reinhardtii* [128], and the engineered 16 kDa N-Luc derived from the sea shrimp *Oplophorus gracilirostris,* which uses the coelenterazine analogue furimazine—a much more stable substrate that allows bioluminescent monitoring over longer timeframes [127].

## 4. Future Directions

*Chlamydomonas reinhardtii* remains the only photosynthetic eukaryote species for which DNA transformation is feasible for all three genomes, and the most advanced microalga in terms of molecular tools and know-how for genetic engineering of both the nucleus and the chloroplast. As such, *C. reinhardtii* is now emerging as an exciting new platform for green biotechnologies in which synthetic biology (synbio) approaches are used to make designer strains for light-driven synthesis of novel products. Recent progress in nuclear engineering has seen the development of gene editing methods for targeted gene knockouts [129,130], standardised DNA parts and assembly syntax for nuclear synbio [131], and novel strategies for efficient transgene expression [132,133,134]. When combined with chloroplast engineering, these tools open the door to even more sophisticated engineering programs such as strains in which product synthesis in the chloroplast is under nuclear control, or strains making novel metabolites (e.g., designer terpenoids or lipids) though engineering of both chloroplast- and cytosol-localised biosynthetic pathways [135]. 

To realise this, the chloroplast molecular toolbox needs to be further refined, and a key part of this is the development of new markers and selection methods that allow multigenic engineering with different transgenes targeted to multiple loci or assembled into operons. Currently, almost all reports of engineering the *C. reinhardtii* plastome involve single transgenes, and there are only a couple of reports where two transgenes have been successfully co-transcribed or targeted to separate loci [13]. However, the impressive progress in chloroplast metabolic engineering in plants where, for example, pathways involving six or eleven genes have been constructed for novel isoprenoid synthesis [136,137] highlight the opportunities for future exploitation of the algal chloroplast. 

A second key requirement is the development of new tools for inducible and tuneable regulation of transgenes in the *C. reinhardtii* chloroplast. Once again, the current situation is decidedly limited with most transgenes being expressed constitutively from endogenous promoter and 5′ UTR elements, and only a couple of strategies described for the induction or repression of transgenes [8,138]. More synbio research is required using a range of reporter systems in combination with orthogonal regulatory elements in order to close the complexity gap between established platforms such as *E. coli* and the chloroplast [139]. This will allow the development of synbio devices similar to those available for *E. coli* that allow fine control of engineered pathways and massively-induced synthesis of recombinant products [140].

Finally, the commercial exploitation of *C. reinhardtii* will require further advances in chloroplast engineering to address regulatory and public acceptance issues; in particular, the development of additional markers for engineering wild-type (i.e., phototrophic) strains that are not based on antibiotic selection, and therefore are considered more benign. As discussed in Section 2, the progress and ideas generated in this area by plant chloroplast engineers serve as a useful guide to such developments. Alternatively, the co-integration and recombinase-based methods for marker elimination could be developed for *C. reinhardtii*, allowing the creation of marker-free strains. Not only does this circumvent marker-related issues in the commercialisation of strains, but also removes any metabolic burden that expression of the markers places on the engineered strain. This is becoming increasingly important as small companies, such as Triton Algae Innovations, Microsynbiotix and Axitan, seek to exploit the *C. reinhardtii* chloroplast as a platform for making high-value therapeutics such as vaccines and colostrum proteins [141,142,143]. 

## 5. Conclusion

Chloroplast engineering in the green alga *Chlamydomonas reinhardtii* has shown considerable promise in the production of commercially-valuable proteins and metabolites. Whilst a number of selectable markers and reporter genes are available, there are still notable limitations. Further improvement in these areas is required in order to bring the *C. reinhardtii* chloroplast to the forefront as a new expression platform for the biotechnology industry. 

## Figures and Tables

**Table 1:** Selectable markers for chloroplast engineering. Markers discussed in this review are summarized. Details include the conferred phenotype, and examples of organisms for which the use of the marker has been reported.

Gene	Phenotype	Organism	Reference
*aadA*	Streptomycin and spectinomycin resistance	Various, including *Chlamydomonas reinhardtii*, *Euglena gracilis*, *Haematococcus pluvialis*, *Nicotiana tabacum*, *Arabidopsis thaliana*, *Marchantia polymorpha*	[30,31,32,33,34,35]
*aphA-6*	Kanamycin and amikacin resistance	Various, including *C. reinhardtii*, *N. tabacum*, *Gossypium hirsutum*	[36,37,38]
*aac6-aph2*	Tobramycin resistance	*N. tabacum*	[39]
*cat*	Chloramphenicol resistance	*N. tabacum*, *Cyanidioschyzon merolae*	[40,41]
*ble*	Zeomycin resistance	*Nannochloropsis oceanica*	[42]
*ereB*	Erythromycin resistance	*Dunaliella tertiolecta*	[43]
*nptII*	Kanamycin resistance	*N. tabacum*, *G. hirsutum*	[38,44]
*rrnS* and *rrnL* variants	Resistance to spectinomycin, streptomycin, kanamycin, or erythromycin	*C. reinhardtii*	[45]
*psbA* variants	Resistance to various herbicides, e.g., metribuzin, 3-(3,4-dichlorophenyl)-1,1-dimethylurea (DCMU), phenmedipham	*C. reinhardtii*	[46,47]
*bar*	Phosphinothricin resistance	*N. tabacum*, *P. subcordiformis*	[48,49]
*hemL*	Insensitivity to gabaculine	*N. tabacum*	[50]
*ahaS* ^W492S^	Sulfometuron methyl resistance	*Porphyridium sp.*	[51]
Essential photosynthesis genes e.g., *atpB*, *petB*, *psaB*, *psbA*, *psbH*, *rbcL*, *tscA*	Restored photosynthesis in recipient strain	*C. reinhardtii*	[25,30,52,53,54,55,56,57]
*trnW* ^UCA^	Restored photosynthesis by translational read-through of opal mutation in *psaA-3*	*C. reinhardtii*	[58]
*ARG9*	Rescued arginine prototrophy in an *ARG9* mutant strain	*C. reinhardtii*	[59]
*ptxD*	Ability to use phosphite as a source of phosphorus	*C. reinhardtii*	[60]
*ASA2* variant	Insensitivity to tryptophan analogues	*N. tabacum*	[61]
*dao*	Tolerance to D-alanine and sensitivity to D-valine	*N. tabacum*	[62]
*dsdA*	Resistance to D-serine	*N. tabacum*	[63]
*BADH*	Resistance to betaine aldehyde	*N. tabacum*	[64]
*codA*	Sensitivity to 5-fluorocytosine	*N. tabacum*, *C. reinhardtii*	[65,66]

**Table 2:** Luciferase genes developed as reporters for the *C. reinhardtii* chloroplast.

Gene Name	Origin of Luciferase	Gene Details	Size of Protein (kDa)	Substrate and Co-Factor	λ_MAX_ (nm)	Reference
*rluc*	Renilla coral	Native sequence	36	Coelenterazine	480	[123]
*luxCt*	Vibrio bacterium	Codon optimised gene fusion encoding A and B subunits joined by linker.	78	Decanal, FMNH_2_	490	[124]
*lucCP*	Firefly beetle	Codon optimised	61	Luciferin, ATP	550–570	[125]
